# Effects of three immobilizing drug combinations on ventilation, gas exchange and metabolism in free-living African lions (*Panthera leo*)

**DOI:** 10.1093/conphys/coad059

**Published:** 2023-08-10

**Authors:** Ashleigh Claire Donaldson, Peter Erik Buss, Andrea Fuller, Leith Carl Rodney Meyer

**Affiliations:** Department of Paraclinical Sciences, Faculty of Veterinary Science, University of Pretoria, Soutpan Road, Onderstepoort, Pretoria, Gauteng, South Africa, 0110; Centre for Veterinary Wildlife Research, Faculty of Veterinary Science, University of Pretoria, Soutpan Road, Onderstepoort, Pretoria, Gauteng, South Africa, 0110; Center for Zoo and Wild Animal Health, Copenhagen Zoo, Frederiksberg, Denmark, 2000; Brain Function Research Group, School of Physiology, Faculty of Health Sciences, University of the Witwatersrand, York Road, Parktown, Johannesburg, Gauteng, South Africa, 2193; Centre for Veterinary Wildlife Research, Faculty of Veterinary Science, University of Pretoria, Soutpan Road, Onderstepoort, Pretoria, Gauteng, South Africa, 0110; Veterinary Wildlife Services, South African National Parks, Kruger National Park, Skukuza, Mpumalanga, South Africa, 1350; Department of Production Animal Studies, Faculty of Veterinary Science, University of Pretoria, Soutpan Road, Onderstepoort, Pretoria, Gauteng, South Africa, 0110; Department of Paraclinical Sciences, Faculty of Veterinary Science, University of Pretoria, Soutpan Road, Onderstepoort, Pretoria, Gauteng, South Africa, 0110; Centre for Veterinary Wildlife Research, Faculty of Veterinary Science, University of Pretoria, Soutpan Road, Onderstepoort, Pretoria, Gauteng, South Africa, 0110; Brain Function Research Group, School of Physiology, Faculty of Health Sciences, University of the Witwatersrand, York Road, Parktown, Johannesburg, Gauteng, South Africa, 2193; Department of Paraclinical Sciences, Faculty of Veterinary Science, University of Pretoria, Soutpan Road, Onderstepoort, Pretoria, Gauteng, South Africa, 0110; Centre for Veterinary Wildlife Research, Faculty of Veterinary Science, University of Pretoria, Soutpan Road, Onderstepoort, Pretoria, Gauteng, South Africa, 0110; Brain Function Research Group, School of Physiology, Faculty of Health Sciences, University of the Witwatersrand, York Road, Parktown, Johannesburg, Gauteng, South Africa, 2193

**Keywords:** A-a gradient, butorphanol, hypoxaemia, ketamine, medetomidine

## Abstract

Free-living lions (12 per group) were immobilized with tiletamine-zolazepam-medetomidine (TZM), ketamine-medetomidine (KM), or ketamine-butorphanol-medetomidine (KBM). During immobilization, respiratory, blood gas and acid–base variables were monitored for 30 minutes. Respiratory rates were within expected ranges and remained constant throughout the immobilizations. Ventilation increased in lions over the immobilization period from 27.2 ± 9.5 to 35.1 ± 25.4 L/min (TZM), 26.1 ± 14.3 to 28.4 ± 18.4 L/min (KM) and 23.2 ± 10.8 to 26.7 ± 14.2 L/min (KBM). Tidal volume increased over the immobilization period from 1800 ± 710 to 2380 ± 1930 mL/breath (TZM), 1580 ± 470 to 1640 ± 500 mL/breath (KM) and 1600 ± 730 to 1820 ± 880 mL/breath (KBM). Carbon dioxide production was initially lower in KBM (0.4 ± 0.2 L/min) than in TZM (0.5 ± 0.2 L/min) lions but increased over time in all groups. Oxygen consumption was 0.6 ± 0.2 L/min (TZM), 0.5 ± 0.2 L/min (KM) and 0.5 ± 0.2 L/min (KBM) and remained constant throughout the immobilization period. Initially the partial pressure of arterial oxygen was lower in KBM (74.0 ± 7.8 mmHg) than in TZM (78.5 ± 4.7 mmHg) lions, but increased to within expected range in all groups over time. The partial pressure of arterial carbon dioxide was higher throughout the immobilizations in KBM (34.5 ± 4.2 mmHg) than in TZM (32.6 ± 2.2 mmHg) and KM (32.6 ± 3.8 mmHg) lions. Alveolar-arterial gradients were initially elevated, but decreased over time for all groups, although in KM lions it remained elevated (26.9 ± 10.4 mmHg) above the expected normal. Overall, all three drug combinations caused minor respiratory and metabolic side-effects in the immobilized lions. However, initially hypoxaemia occurred as the drug combinations, and possibly the stress induced by the immobilization procedure, hinder alveoli oxygen gas exchange.

## Teaser

Drug combinations used to anaesthetize lions may negatively affect their respiration. We compared two new drug combinations to the conventional combination and found that although breathing was normal during anaesthesia, all three combinations initially caused low blood oxygen levels.

## Introduction

Respiratory compromise is one of the leading causes of death in immobilized wildlife (Kock and Burroughs, 2021). However, the effects of immobilizing drug combinations on respiration in large felids have not been well studied. Free-living African lions (*Panthera leo*) are routinely immobilized for management purposes. Historically, tiletamine-zolazepam, a N-methyl-D-aspartate (NMDA) antagonist and benzodiazepine drug combination, was favoured for the immobilization of free-living lions (McKenzie, 1993). Although effective, this combination is associated with irregular breathing (e.g. apneusis), resulting in moderate respiratory compromise, reflected as increases in partial pressure of arterial carbon dioxide, and decreases in both pH and partial pressure of arterial oxygen (Lin *et al.,* 1993). Muscle rigidity and spasms with associated increases in metabolism are also common side-effects (Berry, 2015). Medetomidine, an easily reversible α_2_ adrenoreceptor-agonist sedative, is now routinely combined with tiletamine-zolazepam (TZM), and this partially reversible combination is currently a preferred drug combination for immobilizing free-living lions (Fahlman *et al.,* 2005; Jacquier *et al.,* 2006). Minor cardiorespiratory side-effects are associated with the TZM combination in lions (Fahlman *et al.,* 2005; Jacquier *et al.,* 2006), but hypoxaemia has been noted in some carnivores (Caulkett and Cattet, 1997; Deem *et al.,* 1998; Cattet *et al.,* 1999; Caulkett *et al.,* 1999; Stegmann and Jago, 2006), including lion (Fahlman *et al.,* 2005; Jacquier *et al.,* 2006).

Often due to the cost of, and limited access to tiletamine-zolazepam drug formulations, various other drug combinations have been used to immobilize lions, with the primary immobilising drug being ketamine (Smuts *et al.,* 1973). Ketamine, a NMDA antagonist, has been used in the immobilization of captive lions, but its use in free-living individuals is limited by its potency and solubility which limits the amount of the concentrated drug formulation that can be fitted in a single dart. It can be used in combination with other drugs, notably medetomidine, which facilitates the immobilization of large carnivores, including free-living lions (Fyumagwa *et al.,* 2012). Ketamine generally does not cause significant respiratory depression when typical doses are given, and ventilatory responses to hypoxia and carbon dioxide are maintained when ketamine is administered as a sole agent (Berry, 2015). Immobilization with ketamine may be associated with an apneustic respiratory pattern; however, minute ventilation and partial pressure of arterial carbon dioxide typically remain within normal limits (Berry, 2015). Minor respiratory depression and hypoxaemia have been reported with the use of ketamine plus medetomidine (KM) in various carnivores (Smuts *et al.,* 1973; Caulkett *et al.,* 1999; Stegmann and Jago, 2006; Fahlman *et al.,* 2008; Fyumagwa *et al.,* 2012).

Another drug that has been successfully used in combination with various other drugs to immobilize various wild carnivore species is butorphanol (Larsen *et al.,* 2002). Butorphanol is a synthetically derived κ-opioid receptor agonist and μ-opioid receptor antagonist (Wells *et al.,* 2008). Butorphanol produces less respiratory depression than pure μ-receptor agonists, such as morphine, because of a “ceiling effect” that is reached (Hammond *et al.,* 2008). This respiratory depression may be exacerbated when butorphanol is administered along with α_2_-agonists and inhalational anaesthetics. A combination of butorphanol with an α_2_-agonist and, or dissociative anaesthetic, results in synergism and anaesthesia with reduced doses and side-effects compared with using the drugs separately (Bush *et al.,* 2012). A combination of butorphanol, medetomidine and midazolam has been used successfully in the immobilization of various species (Eggers, 2016; Blignaut, 2020) including lion (Wenger *et al.,* 2010), as has a combination of butorphanol, azaperone and medetomidine (Semjonov *et al.,* 2017). The use of a combination of ketamine, butorphanol and medetomidine (KBM) has been reported in smaller wild felids such as serval (*Leptailurus serval*) (Langan *et al.,* 2000; Moresco *et al.,* 2009; Blignaut, 2020) and bobcats (*Lynx rufus*) (Rockhill *et al.,* 2011), with no associated hypoxaemia, but it has not yet been used in large free-living felids.

The aim of our study was to compare the effects of TZM, KM and KBM on ventilation, gas exchange, acid–base balance and metabolism, and determine which combination produced the least adverse effects, when used to immobilize free-living African lions. To achieve these aims, respiratory, acid–base and metabolic variables, including arterial blood gases, were evaluated over a 30-minute period in lions immobilized with each drug combination. We hypothesized that ventilatory, gas exchange and metabolic responses would be better in lions immobilized with KBM than TZM or KM.

## Materials & Methods

### Animals

Free-living African lions (23 females and 13 males) were captured at night in the Kruger National Park, South Africa (24°23′52” S, 31°46′40″ E) between April and July 2021. Elevation of the study site was 266 metres above sea level. The average air temperature during the capture period was 22.6 ± 2.7°C.

### Experimental procedure

Lions were randomly allocated to three groups (12 lions per group), based on the three drug combinations—tiletamine–zolazepam–medetomidine (TZM), ketamine–medetomidine (KM) or ketamine–butorphanol–medetomidine (KBM). Captures were carried out according to the methods outlined in Donaldson *et al.* (2023a, 2023b). The intended drug doses for each group were as follows:

a) TZM—Tiletamine-zolazepam 0.6 mg/kg (500 mg powder reconstituted in the supplied diluent to 100 mg/mL, Zoletil 100, Virbac RSA (Pty) Ltd, Halfway House, South Africa) plus 0.036 mg/kg medetomidine (Metonil 40 mg/mL, Wildlife Pharmaceuticals, White River, South Africa).
b) KM—Ketamine 3.0 mg/kg (Ketamine 1 g reconstituted with sterile water to 200 mg/mL, Kyron Laboratories, Johannesburg, South Africa) plus 0.036 mg/kg medetomidine.
c) KBM—Ketamine 1.2 mg/kg plus butorphanol 0.24 mg/kg (Butonil 50 mg/mL, Wildlife Pharmaceuticals South Africa (Pty) Ltd, South Africa) plus medetomidine 0.036 mg/kg.

The immobilized lion was blindfolded, and thoracic limbs hobbled as a safety precaution, placed onto a vehicle and transported to an area removed from the capture site, where it was placed in left lateral recumbency on a table and instrumented with monitoring devices. Physiological variables were evaluated from 15 minutes (T_0_) after the lion becoming immobilized and being deemed safe to approach and handle and repeated at 10 minutes intervals for a further 30 minutes (T_10_, T_20_ & T_30_).

Body temperature was measured using a calibrated digital thermometer (HI 98509 Checktemp 1, Hanna Instruments, Woonsocket, USA; modified to include a protective probe sheath) with its probe placed 100 mm within the rectum. Respiratory rate (fR) and expired minute ventilation at standard body temperature and pressure (VE_BTPS_) were determined by a PowerLab Exercise Physiology System (ML870B80, ADInstruments, Sydney, NSW, Australia) and measured using the expiratory flow waveform displayed by LabChart 7 (ADInstruments), by placing an airtight mask, with a two-way low resistance high flow valve (2730; Hans Rudolph, Inc., OK, USA), over the external nares and mouth, and redirecting expired air through air flow tubing to a gas mixing chamber (MLA245, ADInstruments) and a respiratory flow head (MLT1000L, ADInstruments) linked to a spirometer module (ML140, ADInstruments). An adapted face mask was used to collect expired air in a manner that mimics the breathing that occurs during normal field immobilizations i.e. where animals are not normally intubated (Ramsay, 2014). The mask was positioned over the lion’s muzzle to minimize dead space, and was rendered airtight by tightly taping a rectal glove to the lions face and the mask to create a tight seal between the lion’s muzzle and the face mask (Supplementary Figure 1). From the gas mixing chamber, expired oxygen and carbon dioxide concentrations were determined using a gas analyser (ML206, ADInstruments). From these variables measured, oxygen consumption (VO_2_) (L/minute), carbon dioxide production (VCO_2_) and respiratory exchange ratio (RER—VCO_2_/VO_2_) were calculated using standard formula in the respiratory module of the LabChart software package (ADInstruments). fR, VE_BTPS_, VO_2_, VCO_2_ and RER were compiled into 1-minute average time bins and analysed retrospectively using LabChart. A calibration syringe (3 L) was used to calibrate the spirometer prior to the start of every immobilization and the gas analyser was calibrated with ambient air. A 22-gauge x 1″ intravascular catheter (Introcan, BBraun Medical Inc., Bethlehem, Pennsylvania, USA) was inserted into a dorsal pedal artery and secured in place, and intra-arterial pressures measured using a transducer (Deltran II, Utah Medical, Midvale, Utah, USA) placed at the level of the sternum and zeroed to atmospheric pressure before being connected to a field-ready intra-arterial blood pressure monitor (IntraTorr, IntraVitals, Coventry, England, UK) (Donaldson *et al.,* 2023b). Arterial blood samples were collected into heparinized 1 mL syringes from the catheterized dorsal pedal artery at T_0_, T_10_, T_20_ and T_30_, and immediately analysed using a portable blood gas analyser (EPOC Reader Blood Analysis and pre-calibrated BGEM3 test cards; Epocal, ON, Canada). Variables measured included pH, partial pressures of arterial oxygen (PaO_2_) and carbon dioxide (PaCO_2_). Base excess and bicarbonate (HCO_3_^−^) were calculated by the EPOC from the measured values. The blood gas values were not temperature corrected as the EPOC uses an algorithm based on human physiology to calculate the temperature corrected values, and the built-in temperature correction formula in the EPOC analyser has not been validated for most species, including lions.

Lions were weighed by suspending them on a stretcher of known weight below a scale (Crane Scale 500kh, Miles Industrial Fasteners & Hardware CC, Benoni, South Africa). Their sex was recorded, and they were aged by studying the eruption sequence of deciduous and permanent teeth and the wear of permanent teeth (Smuts *et al.,* 1978).

At the end of the procedure, butorphanol’s effects were antagonized intramuscularly (i.m.) in the hind thigh with naltrexone (50 mg/mL, Kyron Laboratories) at twice the butorphanol dose (mg) and medetomidine’s effects were antagonized (i.m.) with atipamezole (20 mg/mL, V-Tech (Pty) Ltd, Midrand, South Africa) at 5 times the medetomidine dose (mg). All lions were monitored at the processing site and protected from potential attack by other lions or hyaenas until they were fully recovered and had re-joined the pride.

### Calculations

The expected minute ventilation (VE_EXP_) in a lion prior to immobilization was estimated from body mass using the formula (Bide *et al.,* 1997):

$$ {\mathrm{VE}}_{\mathrm{EXP}}=0.518\ {\mathrm{BM}}^{0.802} $$

Expired minute ventilation at standard body temperature and pressure (VE_BTPS_), was divided by respiratory rate (fR) to calculate tidal volume (VT). Expected tidal volume (VT_EXP_) was calculated using the formula (Carroll, 2007):

$$ {\mathrm{VE}}_{\mathrm{EXP}}{\mathrm{fR}}^{-1} $$

The alveolar—arterial oxygen partial pressure gradient [P(A-a)O_2_] was calculated using the formula (Fenn *et al.,* 1946):

$$ \mathrm{P}\left(\mathrm{A}-\mathrm{a}\right){\mathrm{O}}_2={\mathrm{F}}_{\mathrm{I}}{\mathrm{O}}_2\left({\mathrm{P}}_{\mathrm{b}}-{\mathrm{P}}_{\mathrm{H}2\mathrm{O}}\right)-\left({\mathrm{P}\mathrm{aCO}}_2/\mathrm{RER}\right)-{\mathrm{P}\mathrm{aO}}_2 $$

With RER calculated and determined by the Powerlab system (VCO_2_/VO_2_), inspired oxygen fraction (F_I_O_2_) standardized to 20.9% and barometric pressure (P_b_) measured using the portable blood gas analyser prior to each immobilization.

Alveolar vapour pressure of saturated air (P_H2O_), at a specific body temperature (T_b_), was determined using the formula (Barenbrug, 1947):

$$ {\mathrm{P}}_{\mathrm{H}2\mathrm{O}}=4.58\exp \left[\frac{17.27{\mathrm{T}}_{\mathrm{b}}}{273.3+{\mathrm{T}}_{\mathrm{b}}}\right] $$

Expected partial pressure of arterial oxygen (PaO_2EXP_) was calculated using the formula

$$ {\mathrm{P}\mathrm{aO}}_{2\mathrm{EXP}}={\mathrm{FIO}}_2\left({\mathrm{P}}_{\mathrm{b}}-{\mathrm{P}}_{\mathrm{H}2\mathrm{O}}\right)-\left({\mathrm{P}\mathrm{aCO}}_2/\mathrm{RER}\right)-\mathrm{P}\left(\mathrm{A}-\mathrm{a}\right){\mathrm{O}}_2 $$

with inspired oxygen fraction (F_I_O_2_) standardized to 20.9%, P_H2O_ standardized to 47 mmHg and barometric pressure (P_b_) measured using the portable blood gas analyser prior to each immobilization. PaCO_2_ was standardized to a normal expected range with a minimum of 28 mmHg and a maximum of 37.5 mmHg (Fink and Schoolman, 1963; Herbert and Mitchell, 1971; Middleton *et* al., 1981), and RER was standardized to 0.8 (Gessaman and Nagy, 1988). P(A-a)O_2_ was standardized to a normal expected range with a minimum of 5 mmHg and a maximum of 15 mmHg (King, 2004).

**Figure 1:**
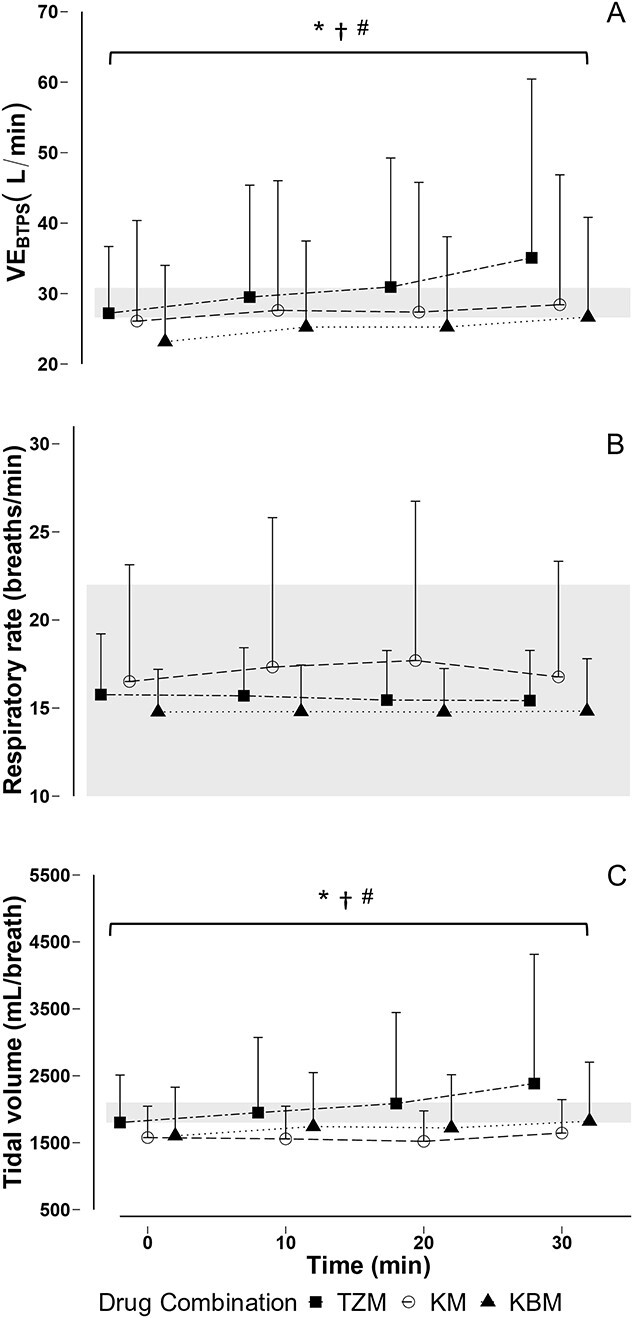
Mean and SD of (A) expired minute ventilation, at standard body temperature and pressure (VE_BTPS_), (B) respiratory rate and (C) tidal volume in free-living African lions (*Panthera leo*) immobilized with tiletamine-zolazepam-medetomidine (TZM), ketamine-medetomidine (KM) or ketamine-butorphanol-medetomidine (KBM). *Note*: Values of each drug combination at specific time points are offset for clarity. Shaded areas represent the following: (A) calculated expected ventilation (26.6–30.8 L/min) (Bide *et al.,* 1997); (B) respiratory rate in awake, unrestrained lions (10–22 breaths/min) (Al-Naji *et al.,* 2019); (C) expected tidal volume, calculated as expected VE_btps_/normal respiratory rate (1800–2100 mL/breath); *P < 0.05 T_30_ vs T_0_ TZM; ^†^*P* < 0.05 T_30_ vs T_0_ KM; ^#^*P* < 0.05 T_30_ vs T_0_ KBM.

Supplementary Table 1 summarizes which variables were measured and which were calculated.

### Statistical analysis

An a priori power analysis for an ANOVA with three groups was conducted in G*Power (Faul *et al.,* 2007) to determine a sufficient sample size using an alpha of 0.05, a power of 0.80, and an effect size of 0.55 to detect a potential difference of 10 mmHg difference in PaO_2_ between treatments. Statistical analysis was performed using Rstudio version 3.6.1 (RStudio: Integrated Development for R. RStudio, PBC, Boston, MA) (R Core Team, 2019). Data are presented as mean ± standard deviation. Physiological data collected over time were compared between groups using a linear mixed effects model. fR, VE_BTPS_, VT, VO_2_, VCO_2_, RER, PaO_2_, PaCO_2_, (P(A-a)O_2_), pH, HCO_3_, base excess and body temperature were designated as response variables. Time, drug combination, sex, age, body mass and body condition were designated as fixed effects and lion ID was designated as the random effect. A temporal autocorrelation term was included in the model (Pinheiro *et al.,* 2021). For each variable, the residuals were calculated, and a Shapiro–Wilk test was used to confirm that the residuals were normally distributed. Residuals for VE_BTPS_ and VT were not normally distributed; thus, the data for these variables were log-transformed, and residuals were re-tested to confirm normality. Significant values were compared using LSMEANS with a Bonferroni correction for multiple pairwise comparisons to determine where differences occurred (Lenth, 2016). After performing a Shapiro–Wilk test to confirm normality of the data, a one-way ANOVA was used to determine if there were differences between mean body mass and age of each group.

**Figure 2:**
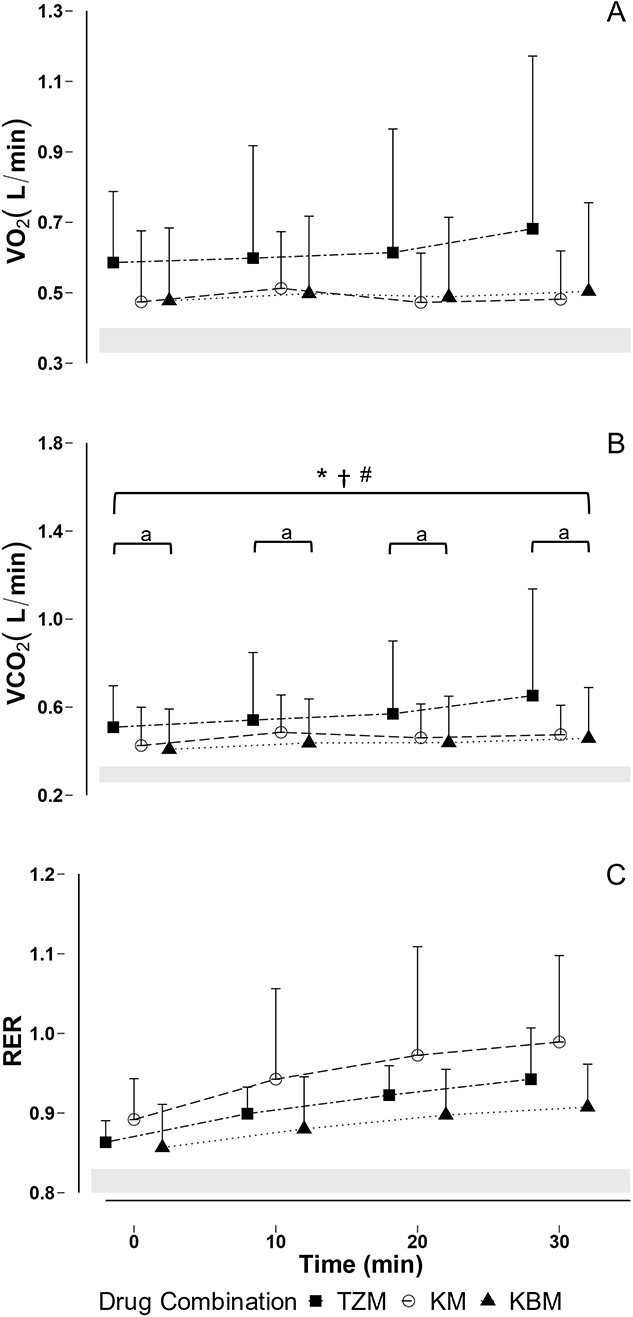
Mean and SD of (A) oxygen consumption (VO_2_), (B) carbon dioxide production (VCO_2_) and (C) respiratory exchange ratio (RER) in free-living African lions (*Panthera leo*) immobilized with tiletamine-zolazepam-medetomidine (TZM), ketamine-medetomidine (KM) or ketamine-butorphanol-medetomidine (KBM). *Note*: Values of each drug combination at specific time points are offset for clarity. Shaded areas represent the following: (A) expected oxygen consumption of free-living African lions at rest (Chassin *et al.,* 1976); (B) expected VCO_2_ of free-living African lions at rest, calculated as expected VO_2_*expected RER (Gessaman and Nagy, 1988); (C) normal respiratory exchange ratio in carnivorous species (Gessaman and Nagy, 1988); *P < 0.05 T_30_ vs T_0_ TZM; ^†^*P* < 0.05 T_30_ vs T_0_ KM; ^#^*P* < 0.05 T_30_ vs T_0_ KBM; ^a^*P* < 0.05 TZM vs KBM.

## Results

The lions had a mean body mass of 143.9 ± 31.6 kg (range 74.0–225.5 kg) and were aged 5.5 ± 2.6 years (range 10 months—12 years). Body mass did not differ between groups at 149.6 ± 21.0 kg (TZM), 136.3 ± 28.7 kg (KM) and 164.0 ± 36.6 kg (KBM) (F = 2.43, *P* = 0.10). There was no difference between the age of lions between the groups, at 6.2 ± 1.8 years (TZM), 5.0 ± 2.9 years (KM) and 5.5 ± 3.0 years (KBM) (F = 0.51, *P* = 0.61).

Mean actual drug doses, determined after weighing the lions, were between 96 to 98% of the intended doses. The mean combined dose of tiletamine-zolazepam administered was 0.58 ± 0.04 mg/kg, mean dose of butorphanol administered was 0.23 ± 0.03 mg/kg, mean dose of ketamine administered was 2.93 ± 0.42 mg/kg (KM) and 1.15 ± 0.13 mg/kg (KBM), and mean dose of medetomidine administered was 0.034 ± 0.003 mg/kg (TZM and KBM) and 0.035 ± 0.005 mg/kg (KM).

The respiratory rate, expired minute ventilation and tidal volume for lions immobilized with the three drug combinations are shown in Figure 1 (and Supplementary Table 2). The respiratory rates of lions at T_0_ did not vary over the 30-minute immobilization period (beta = −0.00, *t* = −0.02, *P* = 0.99) or differ between the drug combinations (beta = −0.65, *t* = −0.32, *P* = 0.75). Over the immobilization the expired minute ventilation did not differ between drug combinations (beta = 9.02, *t* = 1.47, *P* = 0.15), but increased significantly from T_0_ to T_30_ (LSMEANS Bonferroni-adjusted *P =* 0.0125; beta = 4.95, *t* = 3.62, *P* < 0.01). The calculated expected minute ventilation of the study lions did not differ from the measured expired minute ventilation over the entire immobilization period (beta = −15.18, *t* = −0.93, *P* = 0.36). Tidal volumes at T_0_ did not differ between drug combinations (beta = 0.75, *t* = 1.93, *P* = 0.06) and increased significantly over the 30-minute immobilization period (LSMEANS Bonferroni-adjusted *P =* 0.0125; beta = 0.31, *t* = 3.28, *P* = 0.01). Tidal volumes over the entire immobilization period did not differ from calculated expected tidal volumes (beta = −0.13, *t* = −0.51, *P* = 0.62).

The metabolic variables of lions immobilized with the three drug combinations are shown in Figure 2 (and Supplementary Table 3). Oxygen consumption at T_0_ did not differ between drug combinations (beta = −0.11, *t* = −0.96, *P* = 0.99) and remained constant over the 30-minute immobilization period (beta = −0.05, *t* = −1.81, *P* = 0.81). Carbon dioxide production increased significantly over the 30-minute immobilization period in all three groups (LSMEANS Bonferroni-adjusted *P =* 0.0125; beta = 0.09, *t* = 2.78, *P* = 0.01). Carbon dioxide production was significantly lower over the whole immobilization period in the lions immobilized with KBM compared with the lions immobilized with TZM (LSMEANS Bonferroni-adjusted *P =* 0.017; beta = 0.24, *t* = 2.34, *P* = 0.02). Carbon dioxide production of the lions immobilized with KM did not differ from that in either of the other treatments (beta = 0.12, *t* = 1.16, *P* = 0.26). Respiratory exchange ratios for lions at T_0_ did not differ between drug combinations (beta = 0.01, *t* = 0.09, *P* = 0.93) and did not vary over the 30-minute immobilization period (beta = −0.01, *t* = −0.15, *P* = 0.88).

**Figure 3:**
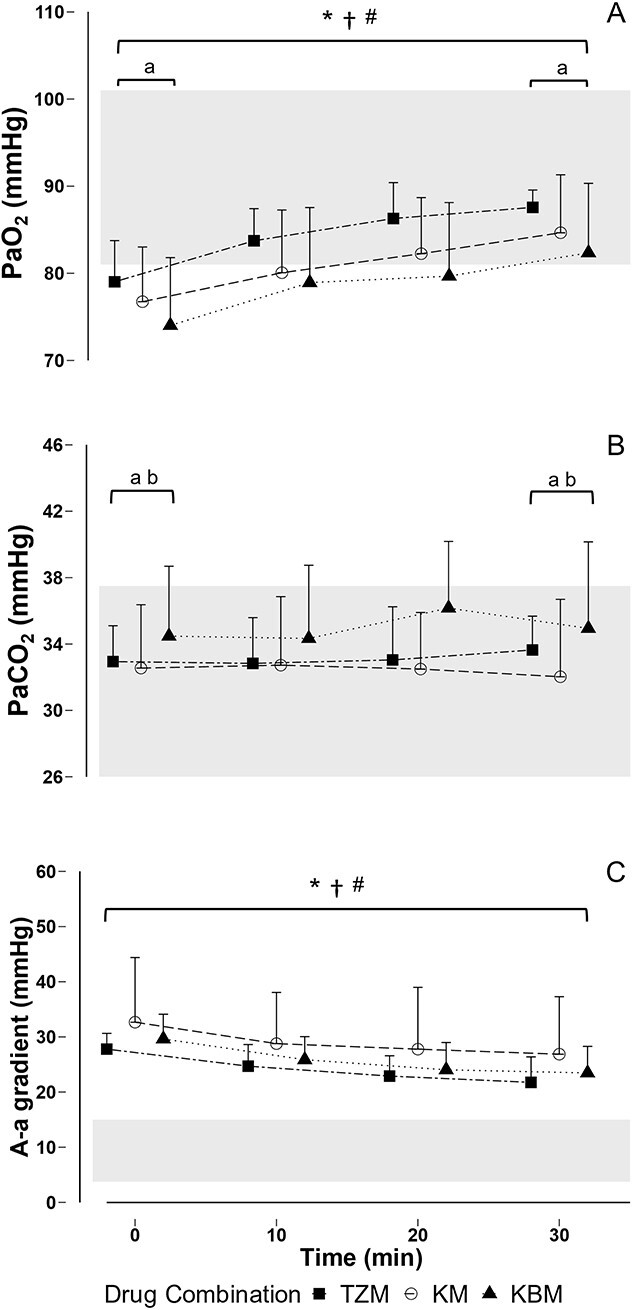
Mean and SD of (A) partial pressure of arterial oxygen (PaO_2_), (B) partial pressure of arterial carbon dioxide (PaCO_2_) and (C) alveolar-arterial (P(A-a)O_2_) gradients African lions (*Panthera leo*) immobilized with tiletamine-zolazepam-medetomidine (TZM), ketamine-medetomidine (KM) or ketamine-butorphanol-medetomidine (KBM). *Note*: Values of each drug combination at specific time points are offset for clarity. Shaded areas represent the following: (A) expected PaO_2_ values at Satara calculated as PaO_2_ = FIO_2_ (P_b_ – P_H__2__O_) – PaCO_2_ - (A – a)O_2_ with FIO_2_ standardized to 20.9% and P_H__2__O_ standardized to 47 mmHg; (B) normal PCO_2_ values in domestic cats (Fink and Schoolman, 1966; Herbert and Mitchell, 1971; Middleton *et* al., 1981); (C) normal A-a gradients in domestic cats (Baylis and Till, 2009); *P < 0.05 T_30_ vs T_0_ TZM; ^†^*P* < 0.05 T_30_ vs T_0_ KM; ^#^*P* < 0.05 T_30_ vs T_0_ KBM; ^a^*P* < 0.05 TZM vs KBM; ^b^*P* < 0.05 KM vs KBM.

Blood gas measurements and calculated alveolar-arterial gradients of lions immobilized with the three drug combinations are shown in Figure 3 (and Supplementary Table 4). Arterial oxygen partial pressure was significantly lower at T_0_ in the lions immobilized with KBM compared with the lions immobilized with TZM (LSMEANS Bonferroni-adjusted *P =* 0.017; beta = 6.34, z = 2.33, *P* = 0.02). Arterial oxygen partial pressure at T_0_ of animals immobilized with KM did not differ from either of the other treatments (beta = 4.07, *t* = 1.46, *P* = 0.16). Arterial oxygen partial pressure increased over the 30-minute immobilization period in all three treatments (LSMEANS Bonferroni-adjusted *P =* 0.0125; beta = 7.92, *t* = 11.98, *P* < 0.01), and remained lower in lions immobilized with KBM than in those immobilized with TZM at T_30_. The calculated expected arterial oxygen partial pressure at the site of capture (266 meters above sea level) was 81-101 mmHg. Arterial carbon dioxide partial pressure was significantly higher at T_0_ in the lions immobilized with KBM compared with the lions immobilized with TZM and KM (LSMEANS Bonferroni-adjusted *P =* 0.017; beta = −2.3, *t* = 5.8, *P* = 0.02). Arterial carbon dioxide partial pressure for lions immobilized with all combinations remained constant over the 30-minute immobilization period (beta = 0.27, *t* = 0.49, *P* = 0.63), with that in lions immobilized with KBM remaining higher than in lions immobilized with TZM or KM. Alveoli oxygen partial pressure for lions immobilized with all combinations remained constant over the 30-minute immobilization period (beta = −2.91, *t* = −2.40, *P* = 1.00) and did not differ between drug combinations (beta = −1.89, *t* = −1.38, *P* = 0.18). Alveolar-arterial gradients for the study lions did not differ between drug combinations (beta = −2.30, *t* = −0.82, *P* = 0.41) and decreased significantly over the 30-minute immobilization period (LSMEANS Bonferroni-adjusted *P =* 0.0125; beta = −5.91, *t* = −7.19, *P* < 0.01).

**Figure 4:**
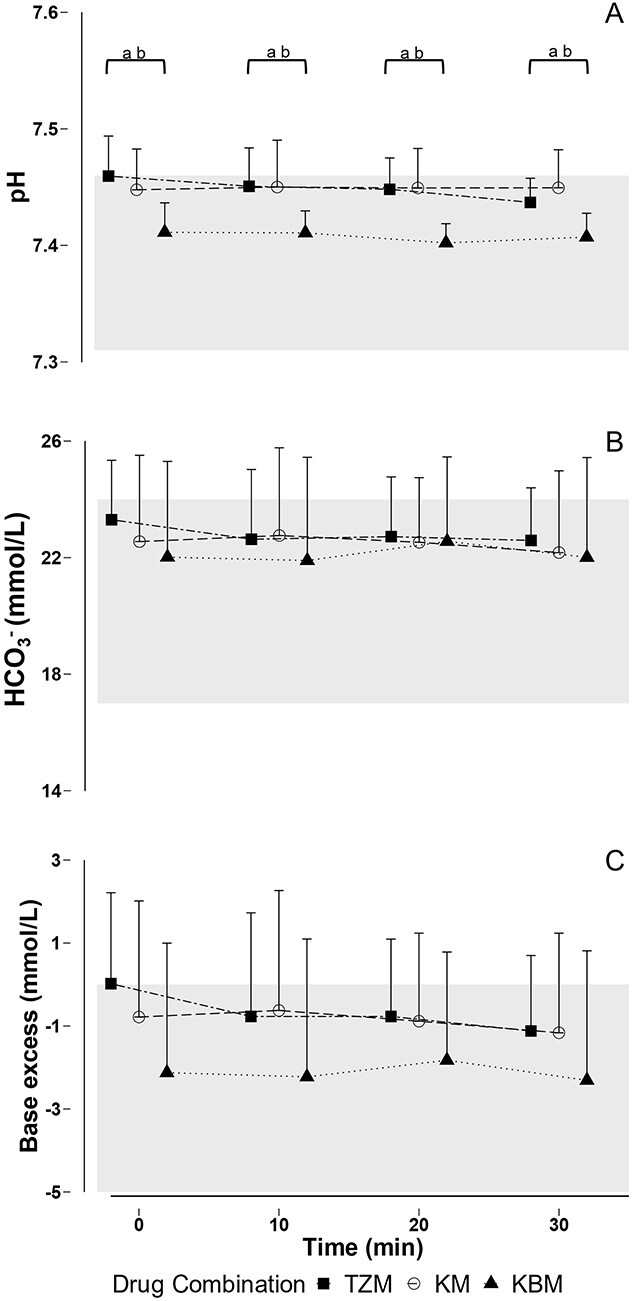
Mean and SD of (A) pH, (B) bicarbonate and (C) base excess in free-living African lions (*Panthera leo*) immobilized with tiletamine-zolazepam-medetomidine (TZM), ketamine-medetomidine (KM) or ketamine-butorphanol-medetomidine (KBM). *Note*: Values of each drug combination at specific time points are offset for clarity. Shaded areas represent the following: (A) normal pH values in domestic cats (Fink and Schoolman, 1966; Herbert and Mitchell, 1971; Middleton *et* al., 1981; (B) normal bicarbonate in domestic cats (Latimer, 2011); (C) normal base excess values in domestic cats (EPOC Veterinary user guide, 2022); ^a^*P* < 0.05 TZM vs KBM; ^b^*P* < 0.05 KM vs KBM.

The acid–base status of lions immobilized with the three drug combinations is shown in Figure 4 (and Supplementary Table 5). pH was significantly lower in animals immobilized with KBM compared with animals immobilized with TZM and KM (LSMEANS Bonferroni-adjusted *P =* 0.017; beta = 0.04, *t* = 3.79, *P* < 0.01). pH in the lions remained constant over the 30-minute immobilization period for all the combinations (beta = 0.01, *t* = 2.68, *P* = 0.25). HCO_3_^−^ of lions did not differ between drug combinations (beta = 1.03, *t* = 0.96, *P* = 0.35) and remained constant over time (beta = −0.45, *t* = −1.18, *P* = 0.24). Base excess of lions did not differ between drug combinations (beta = 1.77, *t* = 1.75, *P* = 0.09) and remained constant over the 30-minute immobilization period (beta = −0.67, *t* = −1.80, *P* = 0.08).

Body temperature did not differ between lions in the three drug combination groups (beta = −0.06, *t* = 0.17, *P* = 0.87) and decreased in all the groups over the 30-minute immobilization period (LSMEANS Bonferroni-adjusted *P =* 0.0125; beta = −0.32, *t* = −8.17, *P* < 0.01) (Supplementary Table 6).

## Discussion

All three drug combinations used in this study produced immobilization without impairing ventilation in lions. Arterial carbon dioxide partial pressure was within normal limits in all groups; however, it was significantly higher in the lions immobilized with KBM compared with the lions immobilized with TZM and KM. Despite adequate ventilation, initially all immobilized lions were mildly hypoxaemic, but the hypoxaemia corrected over the immobilization period. The lions that received KBM initially had the greatest hypoxaemia and took the longest time to become normoxaemic. The initial hypoxaemia can be accounted for by poor pulmonary gas exchange, as indicated by the initially high A-a gradients that improved, but did not entirely correct, over time. Metabolism was elevated throughout the immobilization period in all groups but was lower in lions immobilized with KBM compared with TZM. Correspondingly, lions immobilized with all three drug combinations were mildly hyperthermic throughout the immobilization. Lions immobilized with all three drug combinations had normal acid–base status.

We believe that the strengths of our study include the use of free-living, not captive, lions with limited environmental or temporal variables, i.e. the lions were all immobilized over a short period of time (limiting the influence of seasonal variables) in areas which had very similar vegetation type and prey base (limiting the influences of diet and body condition). An additional strength of the study was the exercise physiology system, which measured and provided novel respiratory and metabolic data in immobilized lions. While reports of physiological measures in immobilized lions are published (Bush *et al.,* 1978; Fahlman *et al.,* 2005; Wenger *et al.,* 2010; Reilly *et al.,* 2014; Semjonov *et al.,* 2017), these studies measured only a subset of respiratory variables. A limitation for all lion research is the lack of reference ranges for healthy, awake lions. We therefore compared respiratory variables to reference ranges for domestic cats, where available. Another limitation of our study was that the mass of the free-living lions could only be estimated before darting, resulting in the lions receiving, on average 2 to 4% less of a drug dose (mg/kg) than the intended dose. However, in all cases the lions became sufficiently immobilized and could be safely handled by the researchers. In addition, resulting physiological effects in each treatment group were similar despite the differences in the immobilizing drugs administered in each combination.

One such variable was respiratory rate, which in awake adult lions at rest is in the range of 10 to 22 breaths per minute (Al-Naji *et al.,* 2019). Immobilized lions in our study had respiratory rates on the higher end of normal (15 to 17 breaths per minute), irrespective of which drug combination was used for immobilization (Figure 1). An elevated respiratory rate during immobilization has been reported with the use of tiletamine-zolazepam (Stirling *et al.,* 1989), medetomidine-butorphanol (Larsen *et al.,* 2002), ketamine (Mitcheltree *et al.,* 1999) and ketamine-xylazine (Mitcheltree *et al.,* 1999) in bears, wolves and mustelids. An elevated respiratory rate can also be attributed to increased body temperature or stress. Hyperthermia is a common occurrence when wildlife species are chemically immobilized due to both drug effects and stress (Fahlman *et al.,* 2008; Meyer *et al.,* 2008; Braud *et al.,* 2019). Body temperatures of lions in this study were elevated (Trethowan *et al.,* 2017) regardless of which immobilizing drug combination was used (Supplementary Table 6). The lions were immobilized on cool autumn or winter nights so any thermal lability that may have been caused by the drugs (Pawson and Forsyth, 2008; Luengos Vidal *et al.,* 2014) unlikely resulted in the observed hyperthermia. As the lions mostly fed on the bait before being darted the increase in body temperatures may have been partly attributed to elevated postprandial metabolism (Trethowan *et al.,* 2017). In addition, increased physical exertion or an excitement-induced stress response may have also contributed to the development of hyperthermia (Fahlman *et al.,* 2008; Hetem *et al.,* 2013; Braud *et al.,* 2019). Competition for food among individual lions in the pride and the resulting excitement of the feeding frenzy could have resulted in increased sympathetic drive (Oka *et al.,* 2001; Bouwknecht *et al.,* 2007; Hetem *et al.,* 2013) and hyperthermia. These potential causes of hyperthermia would not only alter body temperature but possibly also affect respiratory variables.

At T_0_, expired minute ventilation was similar to the expected ventilation, for the lions immobilized with all three drug combinations. Expired minute ventilation increased over time as a result of increases in tidal volume rather than changes in respiratory rate. The changes in expired minute ventilation and tidal volume over time may be attributed to metabolism of the immobilizing drugs (Lin *et al.,* 1993; Lemke, 2004; Wells *et al.,* 2008) leading to less pronounced drug effects and decreased immobilization levels. Medetomidine may cause decreasing respiratory centre chemosensitivity and neurorespiratory drive (Epstein, 2011). Over the course of the immobilization, the medetomidine used in combination to immobilize the lions in all three groups would have been metabolized, possibly lessening the effect on the respiratory centre and allowing for an increase in tidal volume and, in turn, expired minute ventilation. The application of dissociative anaesthetics such as ketamine and tiletamine causes NMDA receptor activity to be inhibited, which can be accompanied by decreased respiratory frequency, and reduced ventilatory responses to hypoxia (Ogawa *et al.,* 1995). Peripheral chemoreceptor activity is also inhibited by opioids such as butorphanol and increased ventilation in response to hypoxia is suppressed (Bailey *et al.,* 2000). Additionally, respiratory depression due to opioids is partly characterized by the suppression of respiratory centres in the brainstem resulting in decreases in minute ventilation and tidal volumes, resulting in hypoxaemia (West, 2016). Similar to medetomidine, the dissociative anaesthetics and the opioid butorphanol used to immobilize the lions in this study would have been metabolized over time, possibly leading to lessening effects and increases in tidal volume and expired minute ventilation.

Because the lions in this study exhibited respiratory rates on the higher end of normal, and adequate ventilation (indicated by PaCO_2_ concentrations within normal range), the initial hypoxaemia was unlikely to have been caused by respiratory depression but possibly rather by an increase in metabolism or inadequate gas exchange. The decrease in drug effects, as the drugs were metabolized and redistributed, likely affected not only ventilation but also whole-body metabolism. Carbon dioxide production and oxygen consumption are measures of metabolic activity in mammals (Takala, 1997). The respiratory exchange ratio for lions in all three groups was similar to the expected ratio of 0.80 to 0.83 in carnivores (Gessaman and Nagy, 1988), and did not exceed a ratio of 1.0 over the immobilization period, indicating that carbon dioxide production never exceeded oxygen consumption, and that respiration remained aerobic throughout the immobilization. The lower VCO_2_ initially in lions immobilized with KBM compared with in those immobilized with TZM may be attributed to better muscle relaxation and lower metabolic demands following KBM administration. Medetomidine induces muscle relaxation (Sinclair, 2003), and butorphanol is combined with ketamine-medetomidine in domestic cats to increase sedation and muscle relaxation (Corona *et al.,* 2020). Conversely, tiletamine-zolazepam and ketamine have been reported to cause muscle rigidity and increased metabolism (Forsyth, 1995; Berry, 2015).

VCO_2_ increased over time in all lions, regardless of the drug combination administered. As the immobilizing drugs were metabolized and redistributed, it is likely that metabolic rate increased resulting in an increase in VCO_2_ as immobilization level decreased. There may have been differences in the metabolism and redistribution of the different drug combinations, which could account for the greater increase in VCO_2_ in lions immobilized with TZM than in those immobilized with KM and KBM. Two lions immobilized with TZM had very high VCO_2_ measurements, which could also account for the larger increase in VCO_2_ in this group. Resting VO_2_ for juvenile lions weighing between 50–57 kg was estimated to be between 0.33 to 0.40 L/min (Chassin *et al.,* 1976), which is lower than the VO_2_ of the lions in all the groups in this study (0.47 to 0.68 L/min). Metabolism scales with body mass, and the resting VO_2_ for larger, adult lions is likely higher than that measured in juveniles and closer to what was calculated in our study. However, the metabolic rate of the animals may be affected by their age as well, so these estimates of what is normal may be incorrect. The increase in VCO_2_ and tendency of RER to increase over the immobilization period without an increase in VO_2_ indicates an increase in whole-body metabolism, but with a change in the metabolic substrate from fats to a mixture of protein and carbohydrates (Gessaman and Nagy, 1988; Birnbaumer *et al.,* 2018; Grandl *et al.,* 2018). Less oxygen is required to aerobically metabolize carbohydrates than fats (Leverve *et al.,* 2007) so, although metabolism increased, the amount of oxygen required to produce energy remained constant.

An increase in PaCO_2_ is expected with an increase in VCO_2_. However, in our study the increase in VE_BTPS_ over the immobilization period possibly explains the PaCO_2_ concentrations remaining constant, as more carbon dioxide was exhaled with increased ventilation. The PaCO_2_ values of the immobilized study lions for all three drug combinations were within expected ranges for awake resting domestic cats (Fink and Schoolman, 1966; Herbert and Mitchell, 1971; Middleton *et* al., 1981). Similar findings of PaCO_2_ values within normal ranges during immobilization have been reported in non-carnivore species (Stemmet *et al.,* 2019).

Hypoxaemia refers to insufficient blood oxygenation to meet metabolic requirements and occurs at sea level when PaO_2_ < 80 mmHg (Read, 2003; Silverstein and Hopper, 2014). Lions immobilized with KBM had lower initial PaO_2_ values (74.0 ± 7.8 mmHg) than those immobilized with TZM (78.4 ± 4.7 mmHg), although all three groups exhibited initial hypoxaemia. The hypoxaemia exhibited by lions in all groups was above the threshold of clinically severe hypoxaemia, defined as PaO_2_ levels below 60 mmHg (King, 2004). Indeed, the PaO_2_ was close to the expected PaO_2_ at the altitude at which the lions were immobilized at, and thus the hypoxaemia could be considered to be mild. In lions immobilized with all three drug combinations, VE_BTPS_ was similar to VE_EXP_ at initial sampling, and PaCO_2_ concentrations were within a normal range, which suggests that the initial hypoxaemia for lions in all three drug combinations was not a result of inadequate ventilation. PaO_2_ in lions immobilized with TZM and KM increased above hypoxaemic levels by T_10_, while KBM immobilized animals only reached this point by T_30_. Lion immobilized with KBM had relatively greater changes in respiration than in those in the other two groups, possibly resulting from the addition of butorphanol. Administration of opioids such as butorphanol may result in decreased minute ventilation and tidal volumes, resulting in hypercapnia and hypoxaemia (West, 2016) and may explain the relatively higher PaCO_2_ (although within a normal range) and lower PaO_2_ in lions immobilized with KBM. As discussed above, the metabolism of the immobilizing drugs over time may have contributed to increases in PaO_2_ as minute ventilation improved. Furthermore, the increase in VE_BTPS_ over time without an increase in VO_2_ could account for some of the improvement of PaO_2_ values over the course of the immobilization.

The resting A-a gradient for lions is unknown, although values below 15 mmHg are normal and anything above 25 mmHg is considered clearly abnormal for domestic cats (Baylis and Till, 2009). A-a gradients for the lions immobilized with all three drug combinations were elevated above the normal domestic cat range at initial sampling, but those in lions immobilized with TZM decreased to below 25 mmHg by T_10_ and those in lions immobilized with KBM decreased to below 25 mmHg by T_20_. Mean A-a gradients of lions immobilized with KM remained above 25 mmHg for the duration of immobilization, although the difference in A-a gradients between groups was not significant due to large standard errors of A-a gradients of lions immobilized with KM. The decrease in A-a gradients of lions in our study over time corresponded to increased PaO_2_ over time. Therefore, the improved PaO_2_ over time could also be accounted for by an improvement in gas exchange.

One possible explanation for the elevated A-a gradients and low PaO_2_ concentrations observed at T_0_ is pulmonary hypertension, brought about by the excitement-induced stress response at capture or through medetomidine’s known effect of causing pulmonary hypertension (Regada, 2013). Pulmonary hypertension can cause pulmonary congestion and oedema, which may hinder gas exchange, and is commonly associated with hypoxaemia. Pulmonary oedema has been reported in domestic cats sedated with α_2_-agonists (Regada, 2013). The hypoxaemia associated with poor gas exchange can also be caused by V/Q mismatch, low diffusion capacity, shunting or low cardiac output (Vodoz *et al.,* 2009), as indicated by the elevated A-a gradients (West, 2016). Ventilation/perfusion mismatches in recumbent animals can be due to lung compression by the body’s mass and a decrease in tidal volume caused by abdominal organs compressing the diaphragm (Morkel *et al.,* 2010; Ambrisko *et al.,* 2017). Furthermore, α_2_-agonists are known to alter pulmonary perfusion (McDonell and Kerr, 2015), causing a ventilation/perfusion mismatch (Rankin, 2015). It is possible that interpulmonary shunting occurred during anaesthesia in the lions; however, with the data that we collected we could not accurately measure shunting.

Impaired gas diffusion, ventilation/perfusion mismatching, and shunting caused by α_2_-agonists and opioids has been described in several species (Caulkett and Arnemo, 2015). When used alone, there is little evidence of α_2_-agonists causing hypoxaemia in healthy domestic cats (Lamont *et al.,* 2001). However, when given along with other sedatives and anaesthetics, the risk of hypoxaemia produced by an α_2_-agonist is increased. Several studies have demonstrated that medetomidine or dexmedetomidine lowered PaO_2_ concentrations to mildly hypoxemic values when combined with ketamine (Ko *et al.,* 2000; Wiese and Muir, 2007; Zeiler *et al.,* 2014; Kim *et al.,* 2021). In these studies animals did not develop hypercapnia, which indicates that ventilation was adequate, and the hypoxaemia observed was likely induced by impaired gas diffusion, ventilation/perfusion mismatching or shunting.

The lack of hypercapnia in lions in our study was also reflected in their normal acid–base status. A normal blood pH for lions at rest has not been described, but the normal pH for healthy domestic cats is 7.34–7.43 (Fink and Schoolman, 1966; Herbert and Mitchell, 1971; Middleton *et* al., 1981). Lions immobilized with all three drug combinations had a pH that was within that range. Although it remained within normal range, pH was lower in lions immobilized with KBM than in those immobilized with TZM or KM. This lower pH was reflected in the higher mean PaCO_2_ in the KBM group. Tiletamine-zolazepam has been reported to cause a metabolic acidosis with respiratory compensation in both captive and free-living lions (Bush *et al.,* 1978; Fahlman *et al.,* 2005). Additionally, when butorphanol, azaperone, and medetomidine are used to immobilize captive lions, metabolic acidosis has been documented (Semjonov *et al.,* 2017). Free-living and captive lions immobilized with ketamine-medetomidine experienced a mixed metabolic and respiratory acidosis (Quandt, 1992). In domestic cats, breakdown of dietary proteins produces acids, which contribute to an inherent metabolic acidosis and normal pH levels that are lower than those of other species (Cook *et al.,* 1996). All felid species are carnivorous, and it is possible that the metabolic acidosis described in lions in previous immobilization studies could have been a misinterpretation of a normal phenomenon as a result of diet (Fahlman *et al.,* 2005).

The doses of immobilizing drugs used in our study were lower than those used previously in other studies (Bush *et al.,* 1978; Fahlman *et al.,* 2005; Jacquier *et al.,* 2006; Wenger *et al.,* 2010; Fyumagwa *et al.,* 2012; Reilly *et al.,* 2014; Semjonov *et al.,* 2017). These lower doses, used in all three drug combinations, appeared to offer an important advantage for the immobilization of lions in that they did not affect the acid–base status and had only minor effects on their respiration. Although the initial hypoxaemia in lions in our study was mild, future studies may consider using supplemental oxygen from the start of immobilization in order to address this issue early on.

## Conclusion

Although some respiratory aberrations caused by immobilization with TZM, KM and KBM were found, respiration in lions was relatively unaffected and animals breathed well throughout the immobilization. However, our study has revealed that it is important to closely monitor blood oxygenation, even in animals that appear to be breathing normally, as immobilization with these drug combinations hindered pulmonary oxygen gas exchange. Initially KBM caused greater hypoxaemia than did TZM and KM, but this hypoxaemia was short-lived and mild, and all lions had clinically acceptable arterial PaO_2_ by the end of the immobilization. Based on our findings, differences in respiratory variables between lions immobilized with each drug combination were minor and no one drug combination provided an overall clinically relevant advantageous effect on ventilation, gas exchange, acid–base status or metabolism in free-living lions.

## Author contributions

LM and PB: conceptualization. AD, AF, LM and PB: methodology. AD, AF, LM and PB: formal analysis and investigation. AF, LM and PB: supervision, technical assistance, and funding. All authors participated in the writing of the manuscript and approved the final manuscript.

## Conflict of interest

The authors have no conflicts of interest to declare.

## Funding

This study was supported by research funding from the Copenhagen Zoo and the Kevin Richardson Foundation.

## Data availability

The data underlying this article are available in the article and in its online supplementary material.

## Supplementary material

Supplementary material is available at *Conservation Physiology* online.

## Supplementary Material

Web_Material_coad059
