# Supplementary material for: Effects of three immobilizing drug combinations on ventilation, gas exchange and metabolism in free-living African lions (Panthera leo)
Source: Conserv Physiol. 2023 Aug 10;11(1):coad059. doi: 10.1093/conphys/coad059 (PMC10416691; doi:10.1093/conphys/coad059)
Supplement: Web_Material_coad059 [file web_material_coad059.pdf]

Supplementary Material

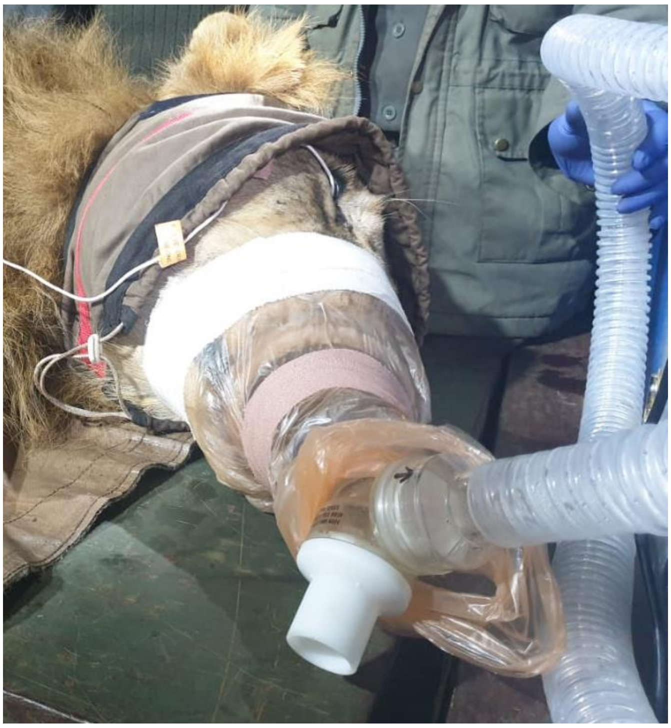

Supplementary Figure 1. The adapted face mask used to collect expired air in free-living African lions (*Panthera leo*) immobilised with tiletamine-zolazepam-medetomidine, ketamine-medetomidine or ketamine-butorphanol-medetomidine. The mask was placed over the muzzle of the lion and made airtight by using a rectal glove gasket seal, which was secured onto the mask and face using tape. Expired air was redirected through air flow tubing to a gas mixing chamber and a respiratory flow head linked to a spirometer module of the PowerLab Exercise Physiology System (ADInstruments).

Supplementary Table 1 Measured and calculated variables from free-living African lions (*Panthera leo*) immobilised with tiletamine-zolazepam-medetomidine (TZM), ketamine-medetomidine (KM) or ketamine-butorphanol-medetomidine (KBM) (n = 12 per drug combination).

Variable	Measured	Calculated
Digital thermometer		
Rectal temperature	✓	
Powerlab[1]		
Respiratory rate (fR)	✓	
Expired minute ventilation at standard body temperature and pressure (VE_{BTPS})	✓	
Oxygen consumption (VO_2)		✓
Carbon dioxide production (VCO_2)		✓
Respiratory exchange ratio (RER)		✓
EPOC blood gas analyser[2]		
pH	✓	
Partial pressure of arterial oxygen (PaO_2)	✓	
Partial pressure of arterial carbon dioxide ($PaCO_2$)	✓	
Base excess		✓
Bicarbonate (HCO_3^-)		✓
Barometric pressure (P_b)	✓	
Haemoglobin concentration ([Hb])		✓
Haemoglobin saturation of oxygen in arterial blood (SaO_2)		✓
Manual calculations		
Expected minute ventilation (VE_{EXP})		✓
Tidal volume (VT)		✓
Expected tidal volume (VT_{EXP})		✓
Alveolar-arterial oxygen partial pressure gradient ($P(A\text{-}a)O_2$)		✓
Alveolar vapor pressure of saturated air at specific body temperature (P_{H_2O})		✓
Expected partial pressure of arterial oxygen (PaO_{2EXP})		✓

1, Calculated variables obtained from the Powerlab system were calculated by LabChart 7 software (ADInstruments)

2, Calculated variables obtained from the EPOC blood gas analyser were calculated using human-derived formulae

Supplementary Table 2 Ventilation at standard body temperature and pressure (VE_{BTPS}), respiratory rate and tidal volume values from free-living African lions (*Panthera leo*) immobilised with tiletamine-zolazepam-medetomidine (TZM), ketamine-medetomidine (KM) or ketamine-butorphanol-medetomidine (KBM) (n = 12 per drug combination).

Sampling time	Distribution	VE_{BTPS} (L/min)			Respiratory rate (breaths/min)			Tidal volume (mL/breath)		
		**TZM**	**KM**	**KBM**	**TZM**	**KM**	**KBM**	**TZM**	**KM**	**KBM**
	Expected mean ± SD	28.7 ± 3.2	26.6 ± 4.5	30.8 ± 5.6				1900 ± 500	1800 ± 600	2100 ± 500
0	Mean	27.2	26.1	23.2	16	17	15	1800	1580	1600
	SD	9.5	14.3	10.8	3	7	2	710	470	730
10	Mean	29.5	27.6	25.2	16	17	15	1950	1560	1740
	SD	15.9	18.4	12.2	3	8	3	1120	490	810
20	Mean	30.9	27.4	25.3	15	18	15	2090	1520	1720
	SD	18.3	18.4	12.8	3	9	2	1360	450	800
30	Mean	35.1*	28.4†	26.7#	15	17	15	2380*	1640†	1820#
	SD	25.4	18.4	14.2	3	7	3	1930	500	880

* Significantly different from measurement at T_0 in TZM group

† Significantly different from measurement at T_0 in KM group

Significantly different from measurement at T_0 in KBM group

[a] Significantly different from animals immobilised with KM at specific sampling point

[b] Significantly different from animals immobilised with TZM at specific sampling point

[c] Significantly different from animals immobilised with KBM at specific sampling point

Supplementary Table 3 Oxygen consumption (VO_2), carbon dioxide production (VCO_2) and respiratory exchange ratio (RER) values from free-living African lions (*Panthera leo*) immobilised with tiletamine-zolazepam-medetomidine (TZM), ketamine-medetomidine (KM) or ketamine-butorphanol-medetomidine (KBM) (n = 12 per drug combination).

Sampling time	Distribution	VO_2 (L/min)			VCO_2 (L/min)			RER		
		**TZM**	**KM**	**KBM**	**TZM**	**KM**	**KBM**	**TZM**	**KM**	**KBM**
0	Mean	0.6	0.5	0.5	0.5[c]	0.4	0.4[b]	0.9	0.9	0.9
	SD	0.2	0.2	0.2	0.2	0.2	0.2	0.0	0.1	0.1
10	Mean	0.6	0.5	0.5	0.5[c]	0.5	0.4$_{b}$	0.9	0.9	0.9
	SD	0.3	0.2	0.2	0.3	0.2	0.2	0.0	0.1	0.1
20	Mean	0.6	0.5	0.5	0.6[c]	0.5	0.4[b]	0.9	1.0	0.9
	SD	0.4	0.1	0.2	0.3	0.2	0.2	0.0	0.1	0.1
30	Mean	0.7	0.5	0.5	0.7[*c]	0.5[*]	0.5[#b]	0.9	1.0	0.9
	SD	0.5	0.1	0.3	0.5	0.1	0.2	0.1	0.1	0.1

* Significantly different from measurement at T_0 in TZM group

† Significantly different from measurement at T_0 in KM group

Significantly different from measurement at T_0 in KBM group

[a] Significantly different from animals immobilised with KM at specific sampling point

[b] Significantly different from animals immobilised with TZM at specific sampling point

[c] Significantly different from animals immobilised with KBM at specific sampling point

Supplementary Table 4 Partial pressure of arterial oxygen (PaO_2) and carbon dioxide ($PaCO_2$) in arterial blood, partial pressure of alveolar oxygen (PAO_2) and alveolar-arterial ($P(A\text{-}a)O_2$) gradient from free-living African lions (*Panthera leo*) immobilised with tiletamine-zolazepam-medetomidine (TZM), ketamine-medetomidine (KM) or ketamine-butorphanol-medetomidine (KBM) (n = 12 per drug combination; *n = 11).

Sampling time	Distribution	PaO_2 (mmHg)			$PaCO_2$ (mmHg)			PAO_2 (mmHg)			($P(A\text{-}a)O_2$) Gradient		
		**TZM**	**KM**	**KBM**	**TZM**	**KM**	**KBM**	**TZM**	**KM**	**KBM**	**TZM**	**KM**	**KBM**
0	Mean	78.5[c]	76.7	74.0[b]	32.6[c]	32.6[c]	34.5[ab]	106.9	109.6	103.7	27.8	32.7	29.6
	SD	4.7	6.3	7.8	2.2	3.8	4.2	2.8	9.1	6.4	2.8	11.7	4.5
10	Mean	82.7[*]	80.1[†]	78.9[#]	32.3	32.7	34.3	108.4	108.8	104.8	24.7[*]	28.8[†]	25.9[#]
	SD	3.7	7.2	8.6	2.8	4.1	4.4	3.3	6.1	6.9	3.9	9.3	4.2
20	Mean	86.2[*]	82.3[†]	79.7[#]	32.7	32.5	36.2	109.1	110.0	103.7	22.9[*]	27.8[†]	24.0[#]
	SD	4.1	6.4	8.5	3.2	3.4	4.0	3.9	6.2	6.1	3.7	11.2	5.0
30	Mean	86.1[*c]	84.7[†]	82.4[#b]	33.3[c]	32.0[c]	34.9[ab]	109.2	114.3	105.7	21.8[*]	26.9[†]	23.5[#]
	SD	2.0	6.7	8.0	2.0	4.7	5.2	3.7	11.5	6.4	4.6	10.4	4.8

* Significantly different from measurement at T_0 in TZM group

† Significantly different from measurement at T_0 in KM group

Significantly different from measurement at T_0 in KBM group

[a] Significantly different from animals immobilised with KM at specific sampling point

[b] Significantly different from animals immobilised with TZM at specific sampling point

[c] Significantly different from animals immobilised with KBM at specific sampling point

Supplementary Table 5 pH, bicarbonate (HCO^{3-}) and base excess of arterial blood from free-living African lions (*Panthera leo*) immobilised with tiletamine-zolazepam-medetomidine (TZM), ketamine-medetomidine (KM) or ketamine-butorphanol-medetomidine (KBM) (n = 12 per drug combination; *n = 11).

Sampling time	Distribution	pH			HCO_3^- (mmol/L)			Base excess (mmol/L)		
		**TZM**	**KM**	**KBM**	**TZM**	**KM**	**KBM**	**TZM**	**KM**	**KBM**
0	Mean	7.46[c]	7.45[c]	7.41[ab]	23.3	22.6	22.0	0.0	-0.8	-2.1
	SD	0.03	0.03	0.03	2.0	3.0	3.3	2.2	2.8	3.1
10	Mean	7.45[c]	7.45[c]	7.41[ab]	22.6	22.8	21.9	-0.8	-0.6	-2.2
	SD	0.03	0.04	0.02	2.4	3.0	3.5	2.5	2.9	3.3
20	Mean	7.45[c]	7.45[c]	7.40[ab]	22.7	22.5	22.6	-0.8	-0.9	-1.8
	SD	0.03	0.03	0.02	2.1	2.2	2.9	1.9	2.1	2.6
30	Mean	7.44[c]	7.45[c]	7.41[ab]	22.6	22.2	22.0	-1.1	-1.2	-2.3
	SD	0.02	0.03	0.02	1.8	2.8	3.4	1.8	2.4	3.1

[a] Significantly different from animals immobilised with KM at specific sampling point

[b] Significantly different from animals immobilised with TZM at specific sampling point

[c] Significantly different from animals immobilised with KBM at specific sampling point

Supplementary Table 6 Body temperatures from free-living African lions (*Panthera leo*0 immobilised with tiletamine-zolazepam-medetomidine (TZM), ketamine-medetomidine (KM) or ketamine-butorphanol-medetomidine (KBM) (n = 12 per drug combination).

Sampling time	Distribution	Body temperature (°C)		
		**TZM**	**KM**	**KBM**
0	Mean	39.5	39.7	39.5
	SD	0.5	1.2	0.5
10	Mean	39.5	39.7	39.4
	SD	0.5	1.3	0.6
20	Mean	39.3*	39.5†	39.3#
	SD	0.5	1.3	0.6
30	Mean	39.2*	39.5†	39.0#
	SD	0.5	1.2	0.6

* Measurement significantly different from measurement at T_0 in TZM group

† Measurement significantly different from measurement at T_0 in KM group

Measurement significantly different from measurement at T_0 in KBM group

[a] Significantly different from animals immobilised with KM at specific sampling point

[b] Significantly different from animals immobilised with TZM at specific sampling point

[c] Significantly different from animals immobilised with KBM at specific sampling point
